# The Role of Inflammation in Age-Related Macular Degeneration

**DOI:** 10.7150/ijbs.49890

**Published:** 2020-09-23

**Authors:** Wei Tan, Jingling Zou, Shigeo Yoshida, Bing Jiang, Yedi Zhou

**Affiliations:** 1 Department of Ophthalmology, The Second Xiangya Hospital, Central South University, Changsha, Hunan 410011, China;; 2 Hunan Clinical Research Center of Ophthalmic Disease, Changsha, Hunan 410011, China;; 3 Department of Ophthalmology, Kurume University School of Medicine, Kurume, Fukuoka 830-0011, Japan

**Keywords:** inflammation, cytokine, leukocyte, age-related macular degeneration

## Abstract

Age-related macular degeneration (AMD) is a blinding eye disease which incidence gradually increases with age. Inflammation participates in AMD pathogenesis, including choroidal neovascularization and geographic atrophy. It is also a kind of self-protective regulation from injury for the eyes. In this review, we described inflammation in AMD pathogenesis, summarized the roles played by inflammation-related cytokines, including pro-inflammatory and anti-inflammatory cytokines, as well as leukocytes (macrophages, dendritic cells, neutrophils, T lymphocytes and B lymphocytes) in the innate or adaptive immunity in AMD. Possible clinical applications such as potential diagnostic biomarkers and anti-inflammatory therapies were also discussed. This review overviews the inflammation as a target of novel effective therapies in treating AMD.

## Introduction

Age-related macular degeneration (AMD), as the name suggests, is an eye disease closely related to aging with an average onset at around 60 years of age, which causes severe vision loss and blindness, especially in developed countries 1-3. The number of population with AMD is expected to be 196 million by 2020, and will increase to 288 million by 2040 4.

There are mainly two types of AMD: dry (also named non-neovascular, non-exudative or atrophic) AMD and wet (also named neovascular or exudative) AMD (nAMD) 5. As the most common type, dry AMD is characterized by the increase of extracellular deposits called drusen, along with advanced-stage geographic atrophy (GA) which is characterized by decreasing of retinal pigment epithelium (RPE) cells, photoreceptors and choroidal capillaries 5, 6. Currently, there is no effective treatment for GA, and the complement cascade is expected to be a potential therapeutic option 7. On the other hand, in patients with wet AMD, which is characterized by choroidal neovascularization (CNV), leading to severe and fast vision impairment, accompanied by hard exudate, leaking fluid or retinal hemorrhage, RPE detachments or develop fibrosis around neovascular tufts 5, 8. Intravitreal injection of anti-vascular endothelial growth factor (VEGF) agents such as ranibizumab 9 and aflibercept 10, have been widely and effectively used worldwide in the clinical treatment of nAMD via targeting CNV. It has been suggested that anti-VEGF therapy significantly improved vision and quality of life for patients with nAMD 11, 12. Nevertheless, about one-third of patients do not get effects from anti-VEGF therapy owing to macular fibrosis or atrophy 13. In addition, there is a heavy demand for repeated intravitreal injections to maintain efficacy 14, 15, which leads to a heavy economic burden. Because of the limitation of anti-VEGF therapies, the development of novel alternative therapies is urgently needed. Some novel molecules were reported to be potentially therapeutic targets, including secretogranin III 16, tenascin-C 17, vitamin D 18, prorenin receptor 19, galactin-1 20, etc. However, further validations of clinical application have not been reported.

Aging participates in the accumulation of oxidative damage, and it is believed that the primary trigger of age-related degenerative diseases is oxidative damage 21. Numerous studies paid attention to the crosstalk between oxidative stress and inflammation. It has been indicated that oxidative stress induces inflammation during the AMD pathological process 22. Pathological oxidative damage contributes to damaged proteins, lipids and DNA, as well as dysfunction of mitochondria, and generates “oxidation-specific epitopes” (such as AGEs and MDA), induced pro-inflammatory responses, and promoted macrophage infiltration and polarization 22, 23. Inflammation caused by tissue damage is considered to be essential in the protective immune response 21. It is well known that chronic inflammation involves many age-related diseases such as cancer 24 and Alzheimer's Disease 25. In this review, we summarize and discuss the role and mechanism(s) of inflammation, as well as inflammatory cytokines and leukocytes in the pathogenesis of AMD.

## Inflammation in AMD pathogenesis

AMD is the consequence of a multifactorial interaction of metabolism, functions, genetics and the environment, and these multiple factors foster a stage conducive for the chronic structural changes in the macular area (choriocapillaries, Bruch's membrane (BM), RPE, photoreceptor) 2, 26. Early signs of AMD contain the appearance of drusen and changes in retinal pigmentation, while advanced stages show CNV or atrophy of photoreceptor cells and RPE 27. Local inflammation leads to drusogenesis, RPE/photoreceptor degeneration, BM disruption and the development of CNV 26. Thus, inflammation is believed to play indispensable roles in the pathogenesis of both dry and wet AMD.

The occurrence of CNV is the main feature of nAMD, which is associated with inflammatory cytokines, complement system activation, and promotion/inhibition of macrophages/microglia 28. Anti-VEGF therapy is mainly used for treating wet AMD, rather than dry AMD. Cytokines such as IL-6 and IP-10 were significantly altered after intravitreal injection of anti-VEGF agents in wet AMD 29. In dry AMD, with the accumulation of lipofuscin and destruction of phagocytic activity of lysosomal enzymes, photoreceptors and RPE cells are damaged. Inflammatory cells release cytokines to attract more inflammatory cells 30. Therefore, it is speculated that inflammation plays different roles in the pathogenesis of wet and dry AMD, respectively.

In 2001, *Hageman et al.* proved that the inflammatory immune response is associated with drusen, ascribed to multiple components found in drusen, including classic acute phase reactants, complement cascade components, etc 31. Besides, it has been demonstrated that RPE and dendritic cells (DCs) play vital roles in drusogenesis. Choroidal DCs are “activated and recruited” by locally injured and/or sublethal damaged RPE cells, related to RPE blebs, fragments, and debris. It can maintain and enhance the local inflammation by multiple mechanisms, such as forming an immune complex, activating complement and choroidal T-cells or phagocytic cells, collectively contributing to the development of AMD 31, 32.

RPE plays a series of indispensable roles in the eye, such as blood-retinal barrier formation, ocular immune privilege establishment, and secretion of soluble immunomodulatory factors that mediate immunogenic inflammation 33, 34. The breakdown of ocular immune tolerance involves blood-retinal barrier, anti-inflammatory and anti-immune proteins, resulting in the specific attack by effector T cells on autoantigens 34, 35. When the blood-ocular barrier is broken, another defense system, called the local ocular immune system, inhibits pathogenic T cells 36. RPE cells play a regulatory role by secreting soluble inhibitory molecules (transforming growth factor (TGF)-β and thrombospondin (TSP)-1) and transforming T cells into regulatory T cells (Tregs) 36, 37. In addition to RPE cells, microglia, DCs and perivascular macrophages also participate in immunomodulatory 38. On the other hand, under the stimulation of inflammatory mediators, such as tumor necrosis factor (TNF)-α, interferon (IFN)-γ and interleukin (IL)-1β, RPE cells produce cytokines and chemokines, including IL-4, -5, -6, -8, -10, -13, -17, IFN-β, IFN-γ, TGF-β, MCP-1 and VEGF. The interaction of pro-inflammatory and anti-inflammatory cytokines ultimately leads to chronic inflammatory responses 39, 40. Inflammatory cytokines can also enhance the secretion of VEGF, which can initiate and cause the pathological CNV and retinal neovascularization of AMD, and macrophages and lymphocytes were found in the active CNV stage 41. Altered expression levels of inflammatory factors were revealed in AMD 42-46. Besides, RPE cells express a series of necessary cytokine receptors such as IL-1R, -4R, -6R, -8RA, -10RB, IFN-AR1, indicated the sensitivity to systemic and retinal inflammatory signals 40. Moreover, RPE can transport nutrients to photoreceptors and dispose waste products, such as the outer segment is detached and then swallowed by RPE before a new outer segment is formed during the renewal of the photoreceptor membranes 33. However, accompanied with the degeneration of RPE cells, photoreceptor cells are gradually and irreversibly destroyed, which leads to vision loss 47. BM is another vital change, which is characterized by increased thickness, basal layer deposits accumulation and/or drusen formation, and there is irregular pigmentation caused by RPE cell hypertrophy, hyperplasia or atrophy. The pathological changes of BM with age further contribute to RPE cell dysfunction and choriocapillaris disorders 38.

Taken together, current evidence indicated that inflammation plays an integral role in the entire pathogenesis of AMD (Figure 2), especially in CNV or GA. We summarize the mechanisms of inflammation-related cytokines and leukocytes, and look forward to getting more inspiration for clinical treatment in AMD.

## Cytokines

In aging eyes, due to the regulation of pro- and anti-inflammatory cytokines by RPE, low-grade chronic inflammation may be induced by these and continue for a long time, and then promote AMD pathogenesis 45. A variety of cytokines have been found to study the relationship between inflammation and the progression of AMD. Regrettably, it is not found that stable trends in different organizations about related cytokines, so we have summarized the present literature about the changes in cytokines in clinical characteristics in Table 1, and their mechanisms in Table 2.

Pro-inflammatory cytokines include IL-1β, IL-2, IL-6, IL-8, IL-12, IL-17, TNF-α, IFN-γ, colony-stimulating factor (CSF) -1 48-50. Inflammasome connects the sensing of pathogen and danger signals with pro-IL-1β activation, and NLR family pyrin domain-containing3 (NLRP3) inflammasome is closely associated with IL-1β maturation. IL-1β can initiate innate immunity related to inflammation, infection and autoimmunity, such as macrophage recruitment, IL-6 activation and chemokine expression modulation 51-53. If the retina was damaged for a long time, the overreactive neurotoxic microglia would release numerous kinds of pro-inflammatory and cytotoxic factors, including IL-1β, further create a pro-inflammatory environment which is beneficial to the recruitment of retinal microglia and exogenous infiltrating monocytes, and eventually result in progressive photoreceptor degeneration 53, 54. Evidence has been accumulated that IL-1α induced inflammasome increases the susceptibility of RPE cell to cytotoxicity mediated by photooxidative damage 55. On the other hand, IL-1β is also known as a pro-angiogenic factor through stimulating VEGF secretion. IL-1 receptor antagonist (IL-1Ra) treatment that inhibits IL-1β significantly suppressed CNV 56-58. IL-2 participates in RPE cell migration, extracellular matrix (ECM) synthesis, TGF-β2 expression, indicating that IL-2 makes a constructive effect on the fibrosis of macular degeneration 59. IL-6 is a key mediator for promoting subretinal fibrosis, which is considered as an injured repair in damaged organs 60, and serum IL-6 level correlates with GA 61. Besides, it has been reported that IL-6 receptor-mediated activation of STAT3 promotes CNV, and the level of IL-6 is associated with the size and activity of CNV in the aqueous humor of AMD patients 60-62. In the pathological mechanism of AMD, intracellular calcium mobilization, C-reactive protein (CRP) and 25-OH are able to induce IL-8 production and secretion. IL-8 participates in acute and chronic inflammation and has a potent proangiogenic ability. IL-8 causes tissue destruction by further attracting neutrophils and neutrophil-mediated inflammation 63-65. TNF-α promotes CNV formation via upregulating VEGF production through reactive oxygen species (ROS)-dependent β-catenin activation, while the treatment of anti-TNF-α can reduce the size and leakage of CNV in mice 66, 67. A recent study indicated that the TNF level is negatively related to the level of bone morphogenetic protein-4 in CNV lesions, which was increased in the dry AMD and decreased in the wet AMD. Moreover, the reduced level of bone morphogenetic protein-4 by TNF may promote the angiogenic environment of the active CNV lesion 68. Besides, it is found that CSF-1 receptor inhibitor PLX5622 treatment can greatly reduce retinal microglia and the CNV lesion size in mice 69. As another pro-inflammatory cytokine, IL-12 can activate T cells and NK cells, thereby inducing Th1-related inflammation related to wet AMD 70. Interestingly, IL-12 may act as an important anti-angiogenic factor to suppress CNV 70. Likewise, IFN-β therapy can limit microglia/macrophage activation, vessel leakage and the development of CNV in the laser-damage model of nAMD 71. IFN-β and IFN-γ have an antagonistic effect 72. IFN-γ promotes the inflammatory response through the activation of pro-inflammatory cytokines and chemokines, then recruits immune cells like macrophages and T cells 72. IFN-γ is also beneficial to the polarization of M1 macrophages, and synergistically increases the production of IL-6. Moreover, IFN-γ also restrains immune cells associated with autoimmune response and up-regulates anti-inflammatory factors 72. In the pathological process of AMD, blocking IFN-γ may weaken the protective effect of Th2 response, thereby strengthen the destruction of Th1 cells 72. Besides, it has been revealed that IFN-γ induces VEGF secretion in RPE cells, and the progression involves the activation of the Phosphoinositide 3-kinase (PI-3K)/mammalian target of rapamycin (mTOR)/ translational pathway 73.

As a Th17 cytokine, IL-17 has a beneficial effect on inflammation of CNV lesions, through producing γδT cells to strengthen the immune response and probably in a C5a-dependent manner 50. It is indicated that the C5a enhanced secretion of Th17 cytokines from CD4+ T cells and is possibly involved in nAMD 74. Meanwhile, IL-17 contributes to CNV pathogenesis and the effective IL-17 is mainly produced by γδT cells, rather than Th17 cells in the ocular lesions 75. IL-17 is also involved in cell migration and tube formation, thereby exerting angiogenesis effect on choroidal endothelial cells (CECs) via PI3K-Rac1 and RhoA-mediated actin cytoskeleton remodeling 76. IL-17A causes the death of RPE cells 77, and activates IL-1β via NLRP3 in RPE 78. Another study demonstrated that IL-23 is able to induce IL-17 production from γδT cells 75.

On the other hand, anti-inflammatory cytokines include IL-4, IL-10, IL-13 and TGF-β 79. IL-4, as a Th2 cytokine, directly drives macrophage soluble fms-like tyrosine kinase 1 (sFlt-1) secretion in Arg-1+ macrophages, and inhibits angiogenesis 80. IL-10 and its downstream STAT3 signaling activation are major regulators of the aging macrophages mainly toward M2 phenotype, and promote ocular angiogenesis 81. In IL-10¯/¯ mice, CNV is significantly reduced 82. *Matsumura et al.* suggested that the pretreatment of low-dose lipopolysaccharide (LPS) inhibited CNV formation through IL-10 secreted by macrophages 83. Exogenous HSP70 induces IL-10 production via both TLR2 and TLR4 in RPE cells, thereby attenuates the formation of subretinal fibrosis 84. IL-13, mainly produced by Th2 cells and monocytes/macrophages, suppressed ARPE-19 cell proliferation in vitro and promoted epithelial-mesenchymal transition (EMT) 85. The higher level of IL-13 is presented in aqueous humor of AMD 85.

TGF-β is an important promoter of immune homeostasis and tolerance, which inhibits the expansion and function of many components of the immune response 86. TGF-β superfamily members involves angiogenesis, inflammatory reactions, vascular fibrosis, immune responses and crosstalk with other signaling pathways in AMD pathogenesis 87. TGF-β plays a vital role in the formation and development of CNV by Smad2/3-VEGF/TNF-α signaling pathway in wet AMD 88. Interestingly, other studies indicated that deficient of TGF-β signaling leads to retinal degeneration and exacerbates CNV 89, 90. Furthermore, TGF-β promotes the EMT of RPE cells, induces subretinal fibrosis and production of IL-6 60.

Subretinal fibrosis is a clinical manifestation of later period of nAMD 91, which is a wound healing response after CNV, together with the damage of photoreceptors, RPE and choroidal blood vessels, causing irreparable visual impairment 19. Cellular and ECM constituents, and the growth factor mediated EMT act as important roles in the RPE and the complex signaling networks of fibrosis in AMD 28. There are two important processes, including EMT and endothelial-mesenchymal transition (EndMT), and TGF-β is the main regulator and the Snail superfamily are key transcription factors 91. Snail superfamily can bind to the DNA promoter region and stimulate the mesenchymal changes, cell migration and proliferation of different epithelial cells, thereby inhibiting the effects of epithelial molecules 92. TGF-β can upregulate the expression of Snail 92, and suppression of TGF-β reduced the size of subretinal fibrosis *in vivo* 93. Moreover, knockdown of both TGF-β2 and Snail suppressed the EMT process of RPE cells more obviously compared to either single gene silencing 94.

In summary, a series of cytokines play constructive roles in the pathogenesis of AMD, including the formation of CNV and subretinal fibrosis. The development of novel targeted therapies could potentially be considered for further investigations.

## Leukocytes

Leukocytes are immune cells that are closely correlated with AMD pathogenesis. Both innate and adaptive immune cells play key roles in AMD.

### Microglia/Macrophages

A hallmark of AMD development is the recruitment of the innate immune cells in the subretinal area with age 95. The resident immune cells are the microglia in the retina, similar to tissue macrophages, which maintain normal retinal function, including the monitor and phagocytosis of damaged cell components 96. Senescent microglia respond slowly to the injury and microglial dysfunction is a key factor in early AMD 95. When retinal microglia migrate to the subretinal space, they may cause obvious changes in RPE cells, including further accumulation of microglia, increasing inflammation in the outer layer of the retina, and contributing to the formation of new blood vessels in wet AMD 97. Besides, activated microglia maybe neurotoxic, and cause the degeneration of photoreceptors, along with phagocytizing dead photoreceptor cells 98. The infiltration of microglia and macrophages to the injured retina, contribute to the development of retinal neovascularization 96.

It is well-known that macrophages are a key modulator of tissue repair, regeneration and fibrosis. There are two major functional subtypes of macrophages, namely classically activated macrophages (M1) and alternatively activated macrophages (M2). M1, or pro-inflammatory macrophages, are anti-tumoral and cause tissue injury. M2, or anti-inflammatory macrophages, facilitate tissue repair and angiogenesis, as well as tumorigenesis and tumor metastasis. However, these two phenotypes can transform into each other as the microenvironment changes 99-101. IL-1β, IL-12, IL-23, IFN-γ, LPS, and TNF-α induce the M1 macrophages that express CCL3, CCL5, CD80, CCR7, and iNOS. M2 macrophages, induced by IL-4, IL-10, IL-13, TGF-β, can express CCL22, CD206, CD163. ROCK signal can determine the polarization of macrophages to M1 and M2 phenotypes, and aging increases ROCK2 signal transduction, leading to the overexpression of pro-angiogenic M2 macrophages 102. *Yang et al.* demonstrate that M1 macrophages participate in the initial stage of CNV, while M2 phenotype plays an important role in the middle and late stages of CNV development and remodeling, thus, M2 is considered to be more important in the progress of CNV 101. However, *Zhou et al.* indicated that M1 macrophages have a more direct effect in suppressing CNV development, and M1 macrophages were primarily present in the RPE-choroid, while M2 were mainly located in the retina 103. Both macrophage recruitment to BM and polarization of resident choroidal macrophages were related to extracellular deposits, including soft drusen and thick, continuous basal laminar deposits 104. In nAMD patients, a large number of macrophages are involved with considerable florid CNV formations in the submacular choroid 105. Activated macrophages are significantly increased in the submacular choroid related to RPE atrophy in GA eyes 105. It is demonstrated that M2 macrophage polarization and CNV formation are induced by chitinase-3-like-1 (CHI3L1) that can also increase VEGFA expression 106. Besides, there are higher levels of phosphorylated signal transducer and activator of transcription3 (pSTAT3) and higher VEGF secretion in monocytes, promoting the development of CNV 107. *Apte et al.* proved that IL-10 suppressed the recruitment of macrophage to neovascular lesions and enhanced CNV formation 82. Macrophages and microglia may be closely related to RPE degeneration. It was found that even if the number of macrophages in the subretinal space is lower, it may contribute to the apoptosis of RPE cells, thereby promoting the development of AMD 108. It was also discovered that local component 1q (C1q) produced by microglia/macrophages plays a role in inflammasome activation and inflammation, and neutralizing effects of C1q may slow retinal atrophy 109.

### Dendritic Cells

DCs, which are effective antigen-presenting cells (APCs), have the special ability to activate B and T lymphocytes. In other words, while DCs are innate immune cells, they are also closely related to the adaptive immune response 110. Without obvious damage, retinal DCs promote homeostasis, but they respond quickly once an injury occurs (the number increases dramatically and supports T cell activation) 111.* Nakai et al.* revealed that DCs have the pro-angiogenic effect in CNV model, and intravenously injected immature DCs, rather than mature DCs, increased CNV size *in vivo* 112. In the case of RPE cell injury, DCs are presented in drusen-related changes in the retina. Furthermore, it has been discovered that autophagy-related dying RPE cells would gradually be engulfed by macrophages, DCs and living RPE cells *in vitro* 113. DCs in choroid may lose the tolerogenic functions and develop into effective APCs, when pro-inflammatory cytokines like GM-CSF, TNF-α or IL-1 were presented without immunomodulatory cytokines such as MCP-1. Moreover, other immune cells can interact with DCs. For instance, macrophages can synergistically promote antigen presentation by DCs, NK cells and DCs can mutually promote activation and maturity, and produce cytokines 114, 115. Accumulating evidence shows that the occurrence of AMD may be the consequence of the dysregulation of choroidal DCs 114.

### Neutrophils

Neutrophils are the frontline effective cells in the innate immune system, with complex biological functions including regulating acute injury and repair, autoimmunity, and chronic inflammation 116. Once neutrophils are recruited, second-wave inflammation occurs, and leads to the recruitment of monocytes/macrophages 117. It can also stimulate T cell activation by expressing MHC class II^118^. A stronger correlation has been shown between nAMD and neutrophil-to-lymphocyte ratio (NLR) elevation compared with healthy controls 119, and NLR is related to disease severity as well as CNV and lesion size 120, 121. Neutrophils are associated with retinal angiogenesis in laser-induced CNV. Neutrophils produce matrix metalloproteinase 9 (MMP-9), which degrades and reshapes the extracellular matrix (a key process of angiogenesis) and destroys the integrity of the RPE barrier 118. Besides, neutrophils cause angiogenesis by producing pro-angiogenic factors such as VEGF and IL-8, VEGF recruit neutrophils which secret more MMP-9 in turn 118. Furthermore, increased infiltration of lipocalin-2 (LCN-2)-positive neutrophils were found in the choroid and retina of patients with early AMD 122. AKT2/nuclear factor-kB (NF-kB)/LCN-2 signaling axis can mediate inflammation activation in AMD 122. Inhibiting AKT2 decreases LCN-2-mediated neutrophil infiltration into the retina and reverses early AMD-like phenotype changes 123.

### T lymphocytes

**Figure 1:**
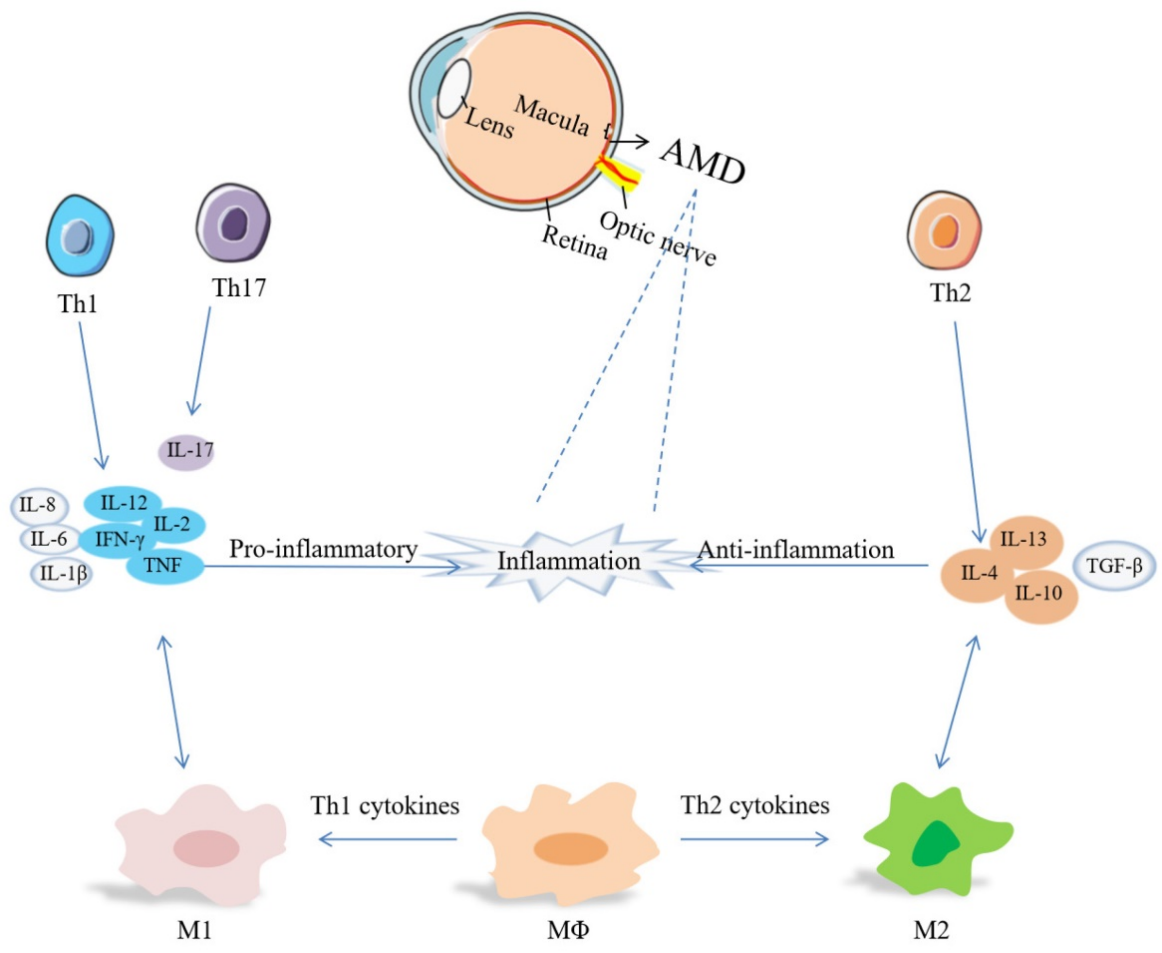
Links between Th cells and macrophages by cytokines in inflammation of AMD. Inflammation includes pro-inflammatory cytokines (IL-1β, IL-2, IL-6, IL-8, IL-12, IL-17, TNF-α, IFN-γ, etc.) and anti-inflammatory cytokines (IL-4, IL-10, IL-13, TGF-β, etc.).

**Figure 2:**
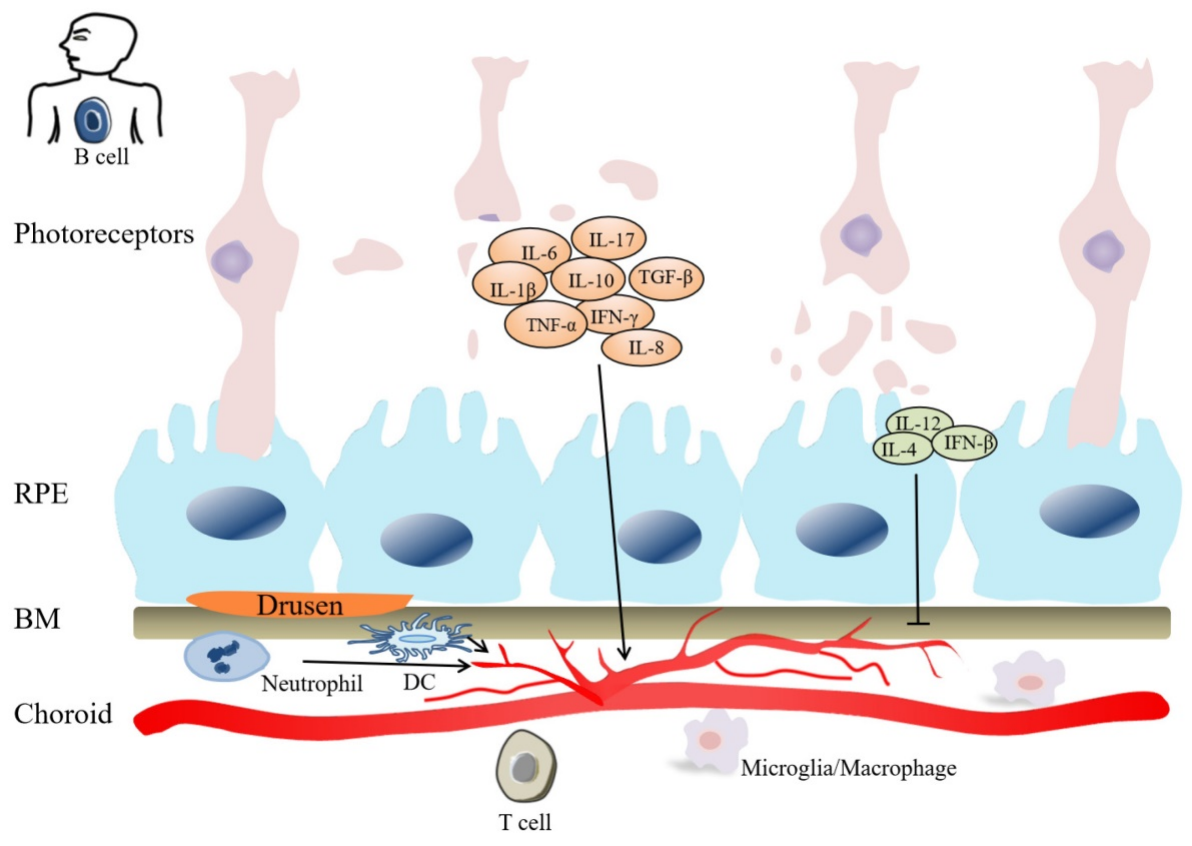
Inflammation plays role in the pathogenesis of AMD. Innate immune cells (macrophages, DCs, Neutrophils) can stimulate adaptive immune cells (B cells and T cells), and participate in CNV pathogenesis. Cytokines, include IL-1β, IL-6, IL-8, IL-10, IL-17, TGF-β, IFN-γ, TNF-α, etc, have angiogenic property. Cytokines, such as IL-4, IL-12, IFN-β, inhibit angiogenesis. In the late stage of AMD, photoreceptor cells are gradually damaged.

T cells are an important part of the adaptive immune system. The evidence of adaptive immunity involved in AMD derives from anti-retinal autoantibodies in AMD patients 124. Th cells activate B cells to produce antibodies, macrophages to damage ingested microbes, and cytotoxic T-cells to destroy infected target cells 125. Carboxyethylpyrrole (CEP) - specific T cells secret pro-inflammatory cytokines, leading to M1 polarization, and link innate immunity and adaptive immunity at the beginning of AMD 124. Th1 cells are associated with IL-2, IL-12, IFN-γ, TNF, while IL-4, IL-10 and IL-13 are involved in Th2 response, and IL-17 is a Th17 cytokine 126. The relevance of Th cells, cytokines and macrophages has been summarized in Figure 1.

Th1 cytokines increased in the vitreous and aqueous humor, while TGF-β can block Th1 cell activation and promote ocular immune tolerance 40. Th1 and Th17 cells can produce pro-inflammatory cytokines, promote the polarization of M1 macrophages, and participate in the process of neovascularization and neurodegeneration 125, 127. In nAMD patients, there are a lower frequency of Th1 cells and CXCR3+CD4+T cells. CXCR3 inhibits angiogenesis, so a lower level of CXCR3 may contribute to angiogenesis and cause CNV formation and growth 125, 128. Th2 and Th17 cells may be involved in the development of subretinal fibrosis 118. Circulating Th1 cells and Th2 cells participate in the pathogenesis of nAMD 129. Patients present a higher level of follicular helper T (Tfh) cells which modulate B cells secreting Ig^130^. It has been recognized that circulating CD56(+) CD28(-) T cells are increased, and CD56(+) is a marker of T cell aging. nAMD is related to T cell immunosenescence 131, 132. *Shi et al.* demonstrated that A2E inhibited the regulatory effects of RPE cells in Th1 cell differentiation by producing IL-1β and suppressing PGE2 133.

### B lymphocytes

The number of B lymphocytes changes with age, which may be because of increased autoimmunity in the elderly population. But there are no significant differences in AMD compared with healthy individuals 134. A study suggested that B cells from advanced AMD patients secret higher levels of antibodies to fight bacterial antigens, especially including IgM, IgG and IgA, and more sensitive to the more diluted concentration of bacterial antigens 135. However, the relationship and mechanisms between B lymphocytes and AMD still remain unclear and further investigations are needed.

## Potential applications in AMD

There are multiple auxiliary diagnostic methods for AMD. Local and systemic inflammatory molecules have been proposed as AMD potential biomarkers, such as CRP, active monocytes, NLR 120, 136-138, but no specific and reliable markers have been found so far.

For early AMD patients, it is suggested to control relevant risk factors, such as quit smoking and keep a balanced and healthy diet 139. The application of anti-angiogenic drugs by blocking VEGF, is a major breakthrough for nAMD patients 140. However, some patients have a poor response. And anti-VEGF therapy cannot change the process of the disease at all, but only resist its effects over time and delays its development 15, 141. Therefore, some scholars have turned their attention to anti-inflammatory therapy which is crucial for AMD pathogenesis. Drugs such as Lampalizumb, Eculizumab, Zimura and Iluvien have initially shown potential effectiveness in clinical trials, and need to be further verified 142-144. As some popular emerging technologies, small interfering RNAs (siRNAs) and clustered regularly interspaced short palindromic repeats (CRISPR)/ CRISPR-associated protein 9 (Cas9) could selectively disrupt the VEGF gene 145-149. Several inflammatory-related cytokines also act on VEGF or VEGF-related mechanisms, so we hypothesized whether can use CRISPR/ Cas9 or siRNAs to target these cytokines and achieved the therapeutic effect on AMD. It is necessary to explore applications of pro- and anti-inflammation in future investigations.

## Conclusion

Although the pathogenesis of AMD is undoubtedly an interaction of multiple factors, significant evidence has emerged implicating inflammation contribute to the development of AMD. RPE releases a large number of inflammatory mediators, contributing to an inflammatory cascade. When the long-term struggle between pro-inflammatory and anti-inflammatory responses eventually loses balance, AMD occurs. Both pro-inflammatory cytokines IL-1β, IL-6, IL-8, IL-12, IL-17, TNF-α, CSF-1, IFN-β and IFN-γ, and anti-inflammatory cytokines IL-4, IL-10 and TGF-β, play important roles in CNV formation through different signaling mechanisms. Similarly, pro-inflammatory cytokines IL-2, IL-6 and anti-inflammatory cytokine IL-10 and TGF-β are involved in the process of fibrosis. Besides, the inflammatory response is inseparable from the accumulation of various inflammatory cells in the eye, mainly innate immune cells such as macrophages, DCs, neutrophils and adaptive immune cells such as T lymphocytes and B lymphocytes. Immune cells can secret cytokines, and are affected by cytokines in turn. Mounting evidence supports the notion that inflammation is involved in AMD. Our review provides an overview of inflammation-related factors that may provide a feasible basis for better treatment options for AMD.

## Figures and Tables

**Table 1:** Expression of different cytokines in samples of patients with AMD.

Cytokine	AMD subtype	Source	Expression
IL-1	Wet AMD	Serum	(IL-1β)↑ 150, (IL-1α+IL-1β)↑^151^
	Wet AMD	Plasma	(IL-1β) ↓^54^, ↑^152^, No change 44
	Wet AMD	Vitreous	(IL-1β)↑^51^
	Wet AMD	Aqueous humor	(IL-1α)↑^153^, ^154^, (IL-1β)No change 153
	Dry AMD	Plasma	(IL-1β)↓^54^
	NA	Aqueous humor	No change 155
IL-2	Wet AMD	Aqueous humor	No change 153, 154, 156
	Dry AMD	Plasma	↓ 54
IL-3	Wet AMD	Aqueous humor	↑ 153
IL-4	Wet AMD	Serum	↑ 151
	Wet AMD	Aqueous humor	No change 153, 154, 156
	NA	Aqueous humor	No change 155
IL-5	Wet AMD	Serum	↑ 151
	Wet AMD	Plasma	↓ 54
	Wet AMD	Aqueous humor	No change 153, 156
	Dry AMD	Plasma	↓ 54
IL-6	Wet AMD Wet AMD	Plasma	↑ 44, 54, 152
		Aqueous humor	↑ 46, 153, ↓ 29, No change 154, 156
	Wet AMD	Serum	No change 150
	Wet AMD	Blood	↑ 157
	Dry AMD	Plasma	↑ 54
	Dry AMD	Serum	↑ 158
	NA	Aqueous humor	No change 155, 159
IL-8	Wet AMD	Aqueous humor	↑ 46, 153, 160, No change 29, 154, 156
	Wet AMD	Plasma	No change 44, 152
	Dry AMD	Serum	↑ 158
	NA	Aqueous humor	No change 155, ↑^159^
IL-10	Wet AMD	Serum	↑ 151
	Wet AMD	Plasma	↓ 54, ↑ 44, 152
	Wet AMD	Aqueous humor	No change 153, 154, 156
	Dry AMD	Plasma	↓ 54
	NA	Aqueous humor	No change 155
IL-12	Wet AMD	Plasma	↓ 54
	Wet AMD	Aqueous humor	(IL-12p40)↑ 153, (IL-12p70)↓^161^, No change 29, 154, (IL-12p70)No change 46, 153, 156
	Dry AMD	Plasma	↓ 54
	NA	Aqueous humor	(IL-12p70)No change 155
IL-13	Wet AMD	Serum	↑ 151
	Wet AMD	Aqueous humor	↓ 156, No change 29, 46, 154
IL-17	Wet AMD	Serum	↑^74^, ^151^
	Wet AMD	Aqueous humor	↓^156^, No change 154
	NA	Aqueous humor	No change 155
	NA	Macular lesion	↑^77^
IL-23	Wet AMD	Aqueous humor	No change 156
GM-CSF	Wet AMD	Plasma	↑ 54
	Wet AMD	Aqueous humor	↓ 162, No change 156
	Dry AMD	Plasma	↑ 54
IFN	Wet AMD Wet AMD	Plasma	(IFN-γ)↑ 54
		Serum	(IFN-β)↑ 163
	Wet AMD	Aqueous humor	(IFN-α+IFN-β+IFN-γ)No change 153
	Dry AMD	Serum	(IFN-β)No change^163^
	Both	Serum	(IFN-α+IFN-γ)No change 163
	NA	Plasma	(IFN-γ)No change 164
	NA	Aqueous humor	(IFN-γ)No change 155
TGF	Wet AMD	Aqueous humor	(TGF-β1)↑ 165, (TGF-β2)↓ 166, (TGF-α+TGF-β)No change 153
	Wet AMD	Vitreous	(TGF-β1)↑ 167
TNF-α	Wet AMD	Blood	No change 157
	Wet AMD	Plasma	No change 168
	Wet AMD	Aqueous humor	↓ 161, No change 46, 160
	Dry AMD	Plasma	↓ 54
	NA	Aqueous humor	No change 155

**Table 2:** The mechanisms of different cytokines in AMD.

Cytokine	Research type	Mechanism(s)
IL-1	In vitro	IL-1α induces inflammasome which increases the sensitivity of RPE cell to cell death mediated by photooxidative damage and the mechanism of cell death becomes pyroptosis 55
	In vivo	IL-1Ra therapy signally suppresses CNV 56, 57
IL-2	In vitro	IL-2 contributes to cell migration, ECM synthesis and TGF-β2 expression via JAK/STAT3 and NF-κB signaling pathways 59
IL-4	In vivo + in vitro	lL-4 suppresses angiogenesis via Arg-1+ macrophage sFlt-1 80
IL-6	In vitro	Proteasome inhibitor MG132 upregulates IL-6 secretion by activating of P38 MAPKs 61
	In vivo + in vitro	IL-6, expressed by activated macrophages, promotes subretinal fibrosis 60 IL-6R-mediated activation of STAT3 contributes to CNV 62
IL-8	In vitro	Intracellular calcium mobilization promotes IL-8 secretion through NF-κB pathway 63 CRP can induce IL-8 expression by multiple pathways 64 25-OH causes IL-8 production through AP-1 and NF-κB pathways 65
IL-10	In vivo + in vitro	IL-10/STAT3 signaling contributes to pathological angiogenesis in senescent macrophages 81 IL-10(-/-) mice can reduce CNV with increased macrophage infiltrates 82 HSP70 induces IL-10 production through TLR2 and TLR4, and reduces subretinal fibrosis 84
	In vivo	CNV is inhibited by low-dose LPS pretreatment through IL-10 secretion by macrophages 83
IL-13	In vitro	IL-13 suppresses ARPE-19 cell proliferation and promotes fibrogenesis 85
IL-17	In vitro	IL-17 involves in choroidal angiogenesis via PI3K-Rac1 and RhoA-mediated actin cytoskeleton remodeling 76
	In vivo + in vitro	IL-17A causes the death of RPE cells by activating Casepase-9 and Casepase-3 77 and activates IL-1β production 78. IL-17 contributes to CNV, and IL-17 mainly produced by γδT cells not Th17 cells in the ocular lesions 75 IL-17 involves inflammation in CNV lesions, through producing γδT cells to strengthen the immune response and probably in a C5a-dependent manner 50.
IFN	In vitro	IFN-γ induces VEGF secretion by PI-3K/mTOR/translational pathway 73
	In vivo	IFN-β therapy weaken microgliosis and macrophage responses in the early AMD and decreased CNV size in the late AMD 71
TGF-β	In vivo	The inhibition of TGF-β/Smad signaling suppresses CNV via down-regulation of VEGF and TNF-α 88 Lack of TGF-β signaling promotes CNV 89, 90
TNF	In vivo +in vitro	BMP4 is down-regulated by TNF by activating JNK pathways in CNV 68 TNF-α promotes CNV by upregulating VEGF secretion via ROS-dependent activation of β-catenin signaling 66
	In vivo	Anti-TNF-α therapy reduces CNV 67

## Abbreviations

AMD: Age-related macular degeneration
nAMD: neovascular AMD
GA: geographic atrophy
RPE: retinal pigment epithelium
CNV: choroidal neovascularization
VEGF: vascular endothelial growth factor
BM: Bruch's membrane
DCs: dendritic cells
TGF: transforming growth factor
TSP: thrombospondin
Tregs: regulatory T cells
TNF: tumor necrosis factor
IFN: interferon
IL: interleukin
CSF: colony-stimulating factor
NLRP3: NLR family pyrin domain-containing3
IL-1Ra: IL-1 receptor antagonist
ECM: extracellular matrix
CRP: C-reactive protein
ROS: reactive oxygen species
PI-3K: Phosphoinositide 3-kinase
mTOR: mammalian target of rapamycin
CECs: choroidal endothelial cells
sFlt-1: soluble fms-like tyrosine kinase 1
LPS: lipopolysaccharide
EMT: epithelial-mesenchymal transition
EndMT: endothelial-mesenchymal transition
CHI3L1: chitinase-3-like-1
pSTAT3: phosphorylated signal transducer and activator of transcription3
C1q: component 1q
APCs: antigen-presenting cells
NLR: neutrophil-to-lymphocyte ratio
MMP-9: matrix metalloproteinase 9
LCN-2: lipocalin-2
NF-kB: nuclear factor-kB
CEP: Carboxyethylpyrrole
Tfh: follicular helper T
siRNAs: small interfering RNAs
CRISPR: clustered regularly interspaced short palindromic repeats
Cas9: CRISPR-associated protein 9

## References

[1] Colijn JM, Buitendijk GHS, Prokofyeva E (2017). Prevalence of Age-Related Macular Degeneration in Europe: The Past and the Future. Ophthalmology.

[2] Al-Zamil WM, Yassin SA (2017). Recent developments in age-related macular degeneration: a review. Clin Interv Aging.

[3] Buitendijk GHS, Rochtchina E, Myers C (2013). Prediction of age-related macular degeneration in the general population: the Three Continent AMD Consortium. Ophthalmology.

[4] Wong WL, Su X, Li X (2014). Global prevalence of age-related macular degeneration and disease burden projection for 2020 and 2040: a systematic review and meta-analysis. Lancet Glob Health.

[5] Mitchell P, Liew G, Gopinath B (2018). Age-related macular degeneration. Lancet.

[6] Yonekawa Y, Miller JW, Kim IK (2015). Age-Related Macular Degeneration: Advances in Management and Diagnosis. J Clin Med.

[7] Boyer DS, Schmidt-Erfurth U, van Lookeren Campagne M (2017). THE PATHOPHYSIOLOGY OF GEOGRAPHIC ATROPHY SECONDARY TO AGE-RELATED MACULAR DEGENERATION AND THE COMPLEMENT PATHWAY AS A THERAPEUTIC TARGET. Retina.

[8] Handa JT, Bowes Rickman C, Dick AD (2019). A systems biology approach towards understanding and treating non-neovascular age-related macular degeneration. Nat Commun.

[9] Frampton JE (2013). Ranibizumab: a review of its use in the treatment of neovascular age-related macular degeneration. Drugs Aging.

[10] Heier JS, Brown DM, Chong V (2012). Intravitreal aflibercept (VEGF trap-eye) in wet age-related macular degeneration. Ophthalmology.

[11] Ferrara N, Adamis AP (2016). Ten years of anti-vascular endothelial growth factor therapy. Nat Rev Drug Discov.

[12] van Lookeren Campagne M, LeCouter J, Yaspan BL (2014). Mechanisms of age-related macular degeneration and therapeutic opportunities. J Pathol.

[13] Little K, Ma JH, Yang N (2018). Myofibroblasts in macular fibrosis secondary to neovascular age-related macular degeneration - the potential sources and molecular cues for their recruitment and activation. EBioMedicine.

[14] Hussain RM, Ciulla TA (2017). Emerging vascular endothelial growth factor antagonists to treat neovascular age-related macular degeneration. Expert Opin Emerg Drugs.

[15] Yang S, Zhao J, Sun X (2016). Resistance to anti-VEGF therapy in neovascular age-related macular degeneration: a comprehensive review. Drug Des Devel Ther.

[16] LeBlanc ME, Wang W, Ji Y (2019). Secretogranin III as a novel target for the therapy of choroidal neovascularization. Exp Eye Res.

[17] Kobayashi Y, Yoshida S, Zhou Y (2016). Tenascin-C secreted by transdifferentiated retinal pigment epithelial cells promotes choroidal neovascularization via integrin alphaV. Lab Invest.

[18] Layana AG, Minnella AM, Garhofer G (2017). Vitamin D and Age-Related Macular Degeneration. Nutrients.

[19] Liu Y, Kanda A, Wu D (2019). Suppression of Choroidal Neovascularization and Fibrosis by a Novel RNAi Therapeutic Agent against (Pro)renin Receptor. Mol Ther Nucleic Acids.

[20] Wu D, Kanda A, Liu Y (2019). Galectin-1 promotes choroidal neovascularization and subretinal fibrosis mediated via epithelial-mesenchymal transition. Faseb j.

[21] Chen M, Xu H (2015). Parainflammation, chronic inflammation, and age-related macular degeneration. J Leukoc Biol.

[22] Datta S, Cano M, Ebrahimi K (2017). The impact of oxidative stress and inflammation on RPE degeneration in non-neovascular AMD. Prog Retin Eye Res.

[23] Du Z, Wu X, Song M (2016). Oxidative damage induces MCP-1 secretion and macrophage aggregation in age-related macular degeneration (AMD). Graefes Arch Clin Exp Ophthalmol.

[24] Diakos CI, Charles KA, McMillan DC (2014). Cancer-related inflammation and treatment effectiveness. Lancet Oncol.

[25] Heppner FL, Ransohoff RM, Becher B (2015). Immune attack: the role of inflammation in Alzheimer disease. Nat Rev Neurosci.

[26] Nowak JZ (2006). Age-related macular degeneration (AMD): pathogenesis and therapy. Pharmacol Rep.

[27] Kanda A, Abecasis G, Swaroop A (2008). Inflammation in the pathogenesis of age-related macular degeneration. Br J Ophthalmol.

[28] Ishikawa K, Kannan R, Hinton DR (2016). Molecular mechanisms of subretinal fibrosis in age-related macular degeneration. Exp Eye Res.

[29] Sato T, Takeuchi M, Karasawa Y (2018). Intraocular inflammatory cytokines in patients with neovascular age-related macular degeneration before and after initiation of intravitreal injection of anti-VEGF inhibitor. Sci Rep.

[30] Damico FM, Gasparin F, Scolari MR (2012). New approaches and potential treatments for dry age-related macular degeneration. Arq Bras Oftalmol.

[31] Hageman GS, Luthert PJ, Victor Chong NH (2001). An integrated hypothesis that considers drusen as biomarkers of immune-mediated processes at the RPE-Bruch's membrane interface in aging and age-related macular degeneration. Prog Retin Eye Res.

[32] Johnson LV, Leitner WP, Staples MK (2001). Complement activation and inflammatory processes in Drusen formation and age related macular degeneration. Exp Eye Res.

[33] Ao J, Wood JP, Chidlow G (2018). Retinal pigment epithelium in the pathogenesis of age-related macular degeneration and photobiomodulation as a potential therapy?. Clin Exp Ophthalmol.

[34] Keino H, Horie S, Sugita S (2018). Immune Privilege and Eye-Derived T-Regulatory Cells. J Immunol Res.

[35] Morohoshi K, Goodwin AM, Ohbayashi M (2009). Autoimmunity in retinal degeneration: autoimmune retinopathy and age-related macular degeneration. J Autoimmun.

[36] Mochizuki M, Sugita S, Kamoi K (2013). Immunological homeostasis of the eye. Prog Retin Eye Res.

[37] Kawazoe Y, Sugita S, Keino H (2012). Retinoic acid from retinal pigment epithelium induces T regulatory cells. Exp Eye Res.

[38] Parmeggiani F, Romano MR, Costagliola C (2012). Mechanism of inflammation in age-related macular degeneration. Mediators Inflamm.

[39] Cheng SC, Huang WC, JH SP (2019). Quercetin Inhibits the Production of IL-1beta-Induced Inflammatory Cytokines and Chemokines in ARPE-19 Cells via the MAPK and NF-kappaB Signaling Pathways. Int J Mol Sci.

[40] Leung KW, Barnstable CJ, Tombran-Tink J (2009). Bacterial endotoxin activates retinal pigment epithelial cells and induces their degeneration through IL-6 and IL-8 autocrine signaling. Mol Immunol.

[41] Nagineni CN, Kommineni VK, William A (2012). Regulation of VEGF expression in human retinal cells by cytokines: implications for the role of inflammation in age-related macular degeneration. J Cell Physiol.

[42] Seddon JM, George S, Rosner B (2005). Progression of age-related macular degeneration: prospective assessment of C-reactive protein, interleukin 6, and other cardiovascular biomarkers. Arch Ophthalmol.

[43] Doyle SL, Ozaki E, Brennan K (2014). IL-18 attenuates experimental choroidal neovascularization as a potential therapy for wet age-related macular degeneration. Sci Transl Med.

[44] Krogh Nielsen M, Subhi Y, Molbech CR (2019). Systemic Levels of Interleukin-6 Correlate With Progression Rate of Geographic Atrophy Secondary to Age-Related Macular Degeneration. Invest Ophthalmol Vis Sci.

[45] Spindler J, Zandi S, Pfister IB (2018). Cytokine profiles in the aqueous humor and serum of patients with dry and treated wet age-related macular degeneration. PLoS One.

[46] Mimura T, Funatsu H, Noma H (2019). Aqueous Humor Levels of Cytokines in Patients with Age-Related Macular Degeneration. Ophthalmologica.

[47] Kivinen N, Koskela A, Kauppinen A (2017). [Pathogenesis of age-related macular degeneration - dialogue between autophagy and inflammasomes]. Duodecim.

[48] Kumari A, Silakari O, Singh RK (2018). Recent advances in colony stimulating factor-1 receptor/c-FMS as an emerging target for various therapeutic implications. Biomed Pharmacother.

[49] Luk HY, Levitt DE, Lee EC (2016). Pro- and anti-inflammatory cytokine responses to a 164-km road cycle ride in a hot environment. Eur J Appl Physiol.

[50] Coughlin B, Schnabolk G, Joseph K (2016). Connecting the innate and adaptive immune responses in mouse choroidal neovascularization via the anaphylatoxin C5a and γδT-cells. Sci Rep.

[51] Zhao M, Bai Y, Xie W (2015). Interleukin-1beta Level Is Increased in Vitreous of Patients with Neovascular Age-Related Macular Degeneration (nAMD) and Polypoidal Choroidal Vasculopathy (PCV). PLoS One.

[52] Wang Y, Hanus JW, Abu-Asab MS (2016). NLRP3 Upregulation in Retinal Pigment Epithelium in Age-Related Macular Degeneration. Int J Mol Sci.

[53] Wooff Y, Man SM, Aggio-Bruce R (2019). IL-1 Family Members Mediate Cell Death, Inflammation and Angiogenesis in Retinal Degenerative Diseases. Front Immunol.

[54] Litwinska Z, Sobus A, Luczkowska K (2019). The Interplay Between Systemic Inflammatory Factors and MicroRNAs in Age-Related Macular Degeneration. Front Aging Neurosci.

[55] Brandstetter C, Patt J, Holz FG (2016). Inflammasome priming increases retinal pigment epithelial cell susceptibility to lipofuscin phototoxicity by changing the cell death mechanism from apoptosis to pyroptosis. J Photochem Photobiol B.

[56] Lavalette S, Raoul W, Houssier M (2011). Interleukin-1beta inhibition prevents choroidal neovascularization and does not exacerbate photoreceptor degeneration. Am J Pathol.

[57] Olson JL, Courtney RJ, Rouhani B (2009). Intravitreal anakinra inhibits choroidal neovascular membrane growth in a rat model. Ocul Immunol Inflamm.

[58] Celkova L, Doyle SL, Campbell M (2015). NLRP3 Inflammasome and Pathobiology in AMD. J Clin Med.

[59] Jing R, Qi T, Wen C (2019). Interleukin-2 induces extracellular matrix synthesis and TGF-beta2 expression in retinal pigment epithelial cells. Dev Growth Differ.

[60] Sato K, Takeda A, Hasegawa E (2018). Interleukin-6 plays a crucial role in the development of subretinal fibrosis in a mouse model. Immunol Med.

[61] Qin T, Gao S (2018). Inhibition of Proteasome Activity Upregulates IL-6 Expression in RPE Cells through the Activation of P38 MAPKs. J Ophthalmol.

[62] Izumi-Nagai K, Nagai N, Ozawa Y (2007). Interleukin-6 receptor-mediated activation of signal transducer and activator of transcription-3 (STAT3) promotes choroidal neovascularization. Am J Pathol.

[63] Yang IH, Wong JH, Chang CM (2015). Involvement of intracellular calcium mobilization in IL-8 activation in human retinal pigment epithelial cells. Invest Ophthalmol Vis Sci.

[64] Wang Y, Bian ZM, Yu WZ (2010). Induction of interleukin-8 gene expression and protein secretion by C-reactive protein in ARPE-19 cells. Exp Eye Res.

[65] Catarino S, Bento CF, Brito A (2012). Regulation of the expression of interleukin-8 induced by 25-hydroxycholesterol in retinal pigment epithelium cells. Acta Ophthalmol.

[66] Wang H, Han X, Wittchen ES (2016). TNF-alpha mediates choroidal neovascularization by upregulating VEGF expression in RPE through ROS-dependent beta-catenin activation. Mol Vis.

[67] Shi X, Semkova I, Muther PS (2006). Inhibition of TNF-alpha reduces laser-induced choroidal neovascularization. Exp Eye Res.

[68] Xu J, Zhu D, He S (2011). Transcriptional regulation of bone morphogenetic protein 4 by tumor necrosis factor and its relationship with age-related macular degeneration. Faseb j.

[69] Schwarzer P, Kokona D, Ebneter A (2020). Effect of Inhibition of Colony-Stimulating Factor 1 Receptor on Choroidal Neovascularization in Mice. Am J Pathol.

[70] Sato T, Takeuchi M, Karasawa Y (2019). Comprehensive expression patterns of inflammatory cytokines in aqueous humor of patients with neovascular age-related macular degeneration. Sci Rep.

[71] Luckoff A, Caramoy A, Scholz R (2016). Interferon-beta signaling in retinal mononuclear phagocytes attenuates pathological neovascularization. EMBO Mol Med.

[72] Jiang K, Cao S, Cui JZ (2013). Immuno-modulatory Effect of IFN-gamma in AMD and its Role as a Possible Target for Therapy. J Clin Exp Ophthalmol.

[73] Liu B, Faia L, Hu M (2010). Pro-angiogenic effect of IFNgamma is dependent on the PI3K/mTOR/translational pathway in human retinal pigmented epithelial cells. Mol Vis.

[74] Liu B, Wei L, Meyerle C (2011). Complement component C5a promotes expression of IL-22 and IL-17 from human T cells and its implication in age-related macular degeneration. J Transl Med.

[75] Hasegawa E, Sonoda KH, Shichita T (2013). IL-23-independent induction of IL-17 from γδT cells and innate lymphoid cells promotes experimental intraocular neovascularization. J Immunol.

[76] Chen Y, Zhong M, Liang L (2014). Interleukin-17 induces angiogenesis in human choroidal endothelial cells in vitro. Invest Ophthalmol Vis Sci.

[77] Ardeljan D, Wang Y, Park S (2014). Interleukin-17 retinotoxicity is prevented by gene transfer of a soluble interleukin-17 receptor acting as a cytokine blocker: implications for age-related macular degeneration. PLoS One.

[78] Zhang S, Yu N, Zhang R (2016). Interleukin-17A Induces IL-1β Secretion From RPE Cells Via the NLRP3 Inflammasome. Invest Ophthalmol Vis Sci.

[79] Boshtam M, Asgary S, Kouhpayeh S (2017). Aptamers Against Pro- and Anti-Inflammatory Cytokines: A Review. Inflammation.

[80] Wu WK, Georgiadis A, Copland DA (2015). IL-4 regulates specific Arg-1(+) macrophage sFlt-1-mediated inhibition of angiogenesis. Am J Pathol.

[81] Nakamura R, Sene A, Santeford A (2015). IL10-driven STAT3 signalling in senescent macrophages promotes pathological eye angiogenesis. Nat Commun.

[82] Apte RS, Richter J, Herndon J (2006). Macrophages inhibit neovascularization in a murine model of age-related macular degeneration. PLoS Med.

[83] Matsumura N, Kamei M, Tsujikawa M (2012). Low-dose lipopolysaccharide pretreatment suppresses choroidal neovascularization via IL-10 induction. PLoS One.

[84] Yang Y, Takeda A, Yoshimura T (2013). IL-10 is significantly involved in HSP70-regulation of experimental subretinal fibrosis. PLoS One.

[85] Fu B, Liu ZL, Zhang H (2017). Interleukin-13 and age-related macular degeneration. Int J Ophthalmol.

[86] Batlle E, Massague J (2019). Transforming Growth Factor-beta Signaling in Immunity and Cancer. Immunity.

[87] Wang K, Li H, Sun R (2019). Emerging roles of transforming growth factor beta signaling in wet age-related macular degeneration. Acta Biochim Biophys Sin (Shanghai).

[88] Wang X, Ma W, Han S (2017). TGF-beta participates choroid neovascularization through Smad2/3-VEGF/TNF-alpha signaling in mice with Laser-induced wet age-related macular degeneration. Sci Rep.

[89] Ma W, Silverman SM, Zhao L (2019). Absence of TGFbeta signaling in retinal microglia induces retinal degeneration and exacerbates choroidal neovascularization. Elife.

[90] Schlecht A, Leimbeck SV, Jagle H (2017). Deletion of Endothelial Transforming Growth Factor-beta Signaling Leads to Choroidal Neovascularization. Am J Pathol.

[91] Shu DY, Butcher E, Saint-Geniez M (2020). EMT and EndMT: Emerging Roles in Age-Related Macular Degeneration. Int J Mol Sci.

[92] Hirasawa M, Noda K, Noda S (2011). Transcriptional factors associated with epithelial-mesenchymal transition in choroidal neovascularization. Mol Vis.

[93] Zhang H, Liu ZL (2012). Transforming growth factor-beta neutralizing antibodies inhibit subretinal fibrosis in a mouse model. Int J Ophthalmol.

[94] Feng Z, Li R, Shi H (2015). Combined silencing of TGF-β2 and Snail genes inhibit epithelial-mesenchymal transition of retinal pigment epithelial cells under hypoxia. Graefes Arch Clin Exp Ophthalmol.

[95] Fletcher EL (2020). Contribution of microglia and monocytes to the development and progression of age related macular degeneration. Ophthalmic Physiol Opt.

[96] Ambati J, Atkinson JP, Gelfand BD (2013). Immunology of age-related macular degeneration. Nat Rev Immunol.

[97] Ma W, Wong WT (2016). Aging Changes in Retinal Microglia and their Relevance to Age-related Retinal Disease. Adv Exp Med Biol.

[98] Telegina DV, Kozhevnikova OS, Kolosova NG (2018). Changes in Retinal Glial Cells with Age and during Development of Age-Related Macular Degeneration. Biochemistry (Mosc).

[99] Wynn TA, Vannella KM (2016). Macrophages in Tissue Repair, Regeneration, and Fibrosis. Immunity.

[100] Chan CC, Ardeljan D (2014). Molecular pathology of macrophages and interleukin-17 in age-related macular degeneration. Adv Exp Med Biol.

[101] Yang Y, Liu F, Tang M (2016). Macrophage polarization in experimental and clinical choroidal neovascularization. Sci Rep.

[102] Zandi S, Nakao S, Chun KH (2015). ROCK-isoform-specific polarization of macrophages associated with age-related macular degeneration. Cell Rep.

[103] Zhou Y, Yoshida S, Kubo Y (2017). Different distributions of M1 and M2 macrophages in a mouse model of laser-induced choroidal neovascularization. Mol Med Rep.

[104] Cherepanoff S, McMenamin P, Gillies MC (2010). Bruch's membrane and choroidal macrophages in early and advanced age-related macular degeneration. Br J Ophthalmol.

[105] McLeod DS, Bhutto I, Edwards MM (2016). Distribution and Quantification of Choroidal Macrophages in Human Eyes With Age-Related Macular Degeneration. Invest Ophthalmol Vis Sci.

[106] Xu N, Bo Q, Shao R (2019). Chitinase-3-Like-1 Promotes M2 Macrophage Differentiation and Induces Choroidal Neovascularization in Neovascular Age-Related Macular Degeneration. Invest Ophthalmol Vis Sci.

[107] Chen M, Lechner J, Zhao J (2016). STAT3 Activation in Circulating Monocytes Contributes to Neovascular Age-Related Macular Degeneration. Curr Mol Med.

[108] Devarajan G, Niven J, Forrester JV (2016). Retinal Pigment Epithelial Cell Apoptosis is Influenced by a Combination of Macrophages and Soluble Mediators Present in Age-Related Macular Degeneration. Curr Eye Res.

[109] Jiao H, Rutar M, Fernando N (2018). Subretinal macrophages produce classical complement activator C1q leading to the progression of focal retinal degeneration. Mol Neurodegener.

[110] Banchereau J, Steinman RM (1998). Dendritic cells and the control of immunity. Nature.

[111] Heuss ND, Lehmann U, Norbury CC (2012). Local activation of dendritic cells alters the pathogenesis of autoimmune disease in the retina. J Immunol.

[112] Nakai K, Fainaru O, Bazinet L (2008). Dendritic cells augment choroidal neovascularization. Invest Ophthalmol Vis Sci.

[113] Szatmari-Toth M, Kristof E, Vereb Z (2016). Clearance of autophagy-associated dying retinal pigment epithelial cells - a possible source for inflammation in age-related macular degeneration. Cell Death Dis.

[114] Forrester JV, Lumsden L, Duncan L (2005). Choroidal dendritic cells require activation to present antigen and resident choroidal macrophages potentiate this response. Br J Ophthalmol.

[115] Lin W, Liu T, Wang B (2019). The role of ocular dendritic cells in uveitis. Immunol Lett.

[116] Liew PX, Kubes P (2019). The Neutrophil's Role During Health and Disease. Physiol Rev.

[117] Prame Kumar K, Nicholls AJ, Wong CHY (2018). Partners in crime: neutrophils and monocytes/macrophages in inflammation and disease. Cell Tissue Res.

[118] Lechner J, Chen M, Hogg RE (2015). Alterations in Circulating Immune Cells in Neovascular Age-Related Macular Degeneration. Sci Rep.

[119] Niazi S, Krogh Nielsen M, Sorensen TL (2019). Neutrophil-to-lymphocyte ratio in age-related macular degeneration: a systematic review and meta-analysis. Acta Ophthalmol.

[120] Ilhan N, Daglioglu MC, Ilhan O (2015). Assessment of Neutrophil/Lymphocyte Ratio in Patients with Age-related Macular Degeneration. Ocul Immunol Inflamm.

[121] Subhi Y, Lykke Sorensen T (2017). New neovascular age-related macular degeneration is associated with systemic leucocyte activity. Acta Ophthalmol.

[122] Ghosh S, Shang P, Yazdankhah M (2017). Activating the AKT2-nuclear factor-kappaB-lipocalin-2 axis elicits an inflammatory response in age-related macular degeneration. J Pathol.

[123] Ghosh S, Padmanabhan A, Vaidya T (2019). Neutrophils homing into the retina trigger pathology in early age-related macular degeneration. Commun Biol.

[124] Cruz-Guilloty F, Saeed AM, Duffort S (2014). T cells and macrophages responding to oxidative damage cooperate in pathogenesis of a mouse model of age-related macular degeneration. PLoS One.

[125] Singh A, Subhi Y, Krogh Nielsen M (2017). Systemic frequencies of T helper 1 and T helper 17 cells in patients with age-related macular degeneration: A case-control study. Sci Rep.

[126] Raphael I, Nalawade S, Eagar TN (2015). T cell subsets and their signature cytokines in autoimmune and inflammatory diseases. Cytokine.

[127] Chen J, Wang W, Li Q (2017). Increased Th1/Th17 Responses Contribute to Low-Grade Inflammation in Age-Related Macular Degeneration. Cell Physiol Biochem.

[128] Falk MK, Singh A, Faber C (2014). Dysregulation of CXCR3 expression on peripheral blood leukocytes in patients with neovascular age-related macular degeneration. Invest Ophthalmol Vis Sci.

[129] Yu Y, Ren XR, Wen F (2016). T-helper-associated cytokines expression by peripheral blood mononuclear cells in patients with polypoidal choroidal vasculopathy and age-related macular degeneration. BMC Ophthalmol.

[130] Wu Q, Liu B, Yuan L (2019). Dysregulations of follicular helper T cells through IL-21 pathway in age-related macular degeneration. Mol Immunol.

[131] Subhi Y, Nielsen MK, Molbech CR (2017). T-cell differentiation and CD56+ levels in polypoidal choroidal vasculopathy and neovascular age-related macular degeneration. Aging (Albany NY).

[132] Faber C, Singh A, Kruger Falk M (2013). Age-related macular degeneration is associated with increased proportion of CD56(+) T cells in peripheral blood. Ophthalmology.

[133] Shi Q, Wang Q, Li J (2015). A2E Suppresses Regulatory Function of RPE Cells in Th1 Cell Differentiation Via Production of IL-1beta and Inhibition of PGE2. Invest Ophthalmol Vis Sci.

[134] Hector SM, Sorensen TL (2017). Circulating monocytes and B-lymphocytes in neovascular age-related macular degeneration. Clin Ophthalmol.

[135] Chen JJ, Han BS, Xu SG (2016). Hypersensitivity toward bacterial stimuli in patients with age-related macular degeneration. Apmis.

[136] Kauppinen A, Paterno JJ, Blasiak J (2016). Inflammation and its role in age-related macular degeneration. Cell Mol Life Sci.

[137] Han X, Ong JS, An J (2020). Using Mendelian randomization to evaluate the causal relationship between serum C-reactive protein levels and age-related macular degeneration. Eur J Epidemiol.

[138] Cousins SW, Espinosa-Heidmann DG, Csaky KG (2004). Monocyte activation in patients with age-related macular degeneration: a biomarker of risk for choroidal neovascularization?. Arch Ophthalmol.

[139] Garcia-Layana A, Cabrera-Lopez F, Garcia-Arumi J (2017). Early and intermediate age-related macular degeneration: update and clinical review. Clin Interv Aging.

[140] Cheung GCM, Lai TYY, Gomi F (2017). Anti-VEGF Therapy for Neovascular AMD and Polypoidal Choroidal Vasculopathy. Asia Pac J Ophthalmol (Phila).

[141] Hernandez-Zimbron LF, Zamora-Alvarado R, Ochoa-De la Paz L (2018). Age-Related Macular Degeneration: New Paradigms for Treatment and Management of AMD. Oxid Med Cell Longev.

[142] Bandello F, Sacconi R, Querques L (2017). Recent advances in the management of dry age-related macular degeneration: A review. F1000Res.

[143] Li H, Chintalapudi SR, Jablonski MM (2017). Current drug and molecular therapies for the treatment of atrophic age-related macular degeneration: phase I to phase III clinical development. Expert Opin Investig Drugs.

[144] Nebbioso M, Lambiase A, Cerini A (2019). Therapeutic Approaches with Intravitreal Injections in Geographic Atrophy Secondary to Age-Related Macular Degeneration: Current Drugs and Potential Molecules. Int J Mol Sci.

[145] Holmgaard A, Alsing S, Askou AL (2019). CRISPR Gene Therapy of the Eye: Targeted Knockout of Vegfa in Mouse Retina by Lentiviral Delivery. Methods Mol Biol.

[146] Kim K, Park SW, Kim JH (2017). Genome surgery using Cas9 ribonucleoproteins for the treatment of age-related macular degeneration. Genome Res.

[147] Wu W, Duan Y, Ma G (2017). AAV-CRISPR/Cas9-Mediated Depletion of VEGFR2 Blocks Angiogenesis In Vitro. Invest Ophthalmol Vis Sci.

[148] Chung SH, Mollhoff IN, Nguyen U (2020). Factors Impacting Efficacy of AAV-Mediated CRISPR-Based Genome Editing for Treatment of Choroidal Neovascularization. Mol Ther Methods Clin Dev.

[149] Garba AO, Mousa SA (2010). Bevasiranib for the treatment of wet, age-related macular degeneration. Ophthalmol Eye Dis.

[150] Budiene B, Liutkeviciene R, Gustiene O (2018). The association of matrix metalloproteinases polymorphisms and interleukins in advanced age-related macular degeneration. Ophthalmic Genet.

[151] Nassar K, Grisanti S, Elfar E (2015). Serum cytokines as biomarkers for age-related macular degeneration. Graefes Arch Clin Exp Ophthalmol.

[152] Subhi Y, Krogh Nielsen M, Molbech CR (2019). Plasma markers of chronic low-grade inflammation in polypoidal choroidal vasculopathy and neovascular age-related macular degeneration. Acta Ophthalmol.

[153] Jonas JB, Tao Y, Neumaier M (2012). Cytokine concentration in aqueous humour of eyes with exudative age-related macular degeneration. Acta Ophthalmol.

[154] Sakurada Y, Nakamura Y, Yoneyama S (2015). Aqueous humor cytokine levels in patients with polypoidal choroidal vasculopathy and neovascular age-related macular degeneration. Ophthalmic Res.

[155] Kramer M, Hasanreisoglu M, Feldman A (2012). Monocyte chemoattractant protein-1 in the aqueous humour of patients with age-related macular degeneration. Clin Exp Ophthalmol.

[156] Zhou H, Zhao X, Yuan M (2020). Comparison of cytokine levels in the aqueous humor of polypoidal choroidal vasculopathy and neovascular age-related macular degeneration patients. BMC Ophthalmol.

[157] Haas P, Kubista KE, Krugluger W (2015). Impact of visceral fat and pro-inflammatory factors on the pathogenesis of age-related macular degeneration. Acta Ophthalmol.

[158] Ambreen F, Ismail M, Qureshi IZ (2015). Association of gene polymorphism with serum levels of inflammatory and angiogenic factors in Pakistani patients with age-related macular degeneration. Mol Vis.

[159] Motohashi R, Noma H, Yasuda K (2017). Dynamics of Inflammatory Factors in Aqueous Humor during Ranibizumab or Aflibercept Treatment for Age-Related Macular Degeneration. Ophthalmic Res.

[160] Ng DS, Yip YW, Bakthavatsalam M (2017). Elevated angiopoietin 2 in aqueous of patients with neovascular age related macular degeneration correlates with disease severity at presentation. Sci Rep.

[161] Rezar-Dreindl S, Sacu S, Eibenberger K (2016). The Intraocular Cytokine Profile and Therapeutic Response in Persistent Neovascular Age-Related Macular Degeneration. Invest Ophthalmol Vis Sci.

[162] Terao N, Koizumi H, Kojima K (2018). Distinct Aqueous Humour Cytokine Profiles of Patients with Pachychoroid Neovasculopathy and Neovascular Age-related Macular Degeneration. Sci Rep.

[163] Afarid M, Azimi A, Malekzadeh M (2019). Evaluation of serum interferons in patients with age-related macular degeneration. J Res Med Sci.

[164] Faber C, Jehs T, Juel HB (2015). Early and exudative age-related macular degeneration is associated with increased plasma levels of soluble TNF receptor II. Acta Ophthalmol.

[165] Tosi GM, Caldi E, Neri G (2017). HTRA1 and TGF-beta1 Concentrations in the Aqueous Humor of Patients With Neovascular Age-Related Macular Degeneration. Invest Ophthalmol Vis Sci.

[166] Tosi GM, Neri G, Caldi E (2018). TGF-beta concentrations and activity are down-regulated in the aqueous humor of patients with neovascular age-related macular degeneration. Sci Rep.

[167] Bai Y, Liang S, Yu W (2014). Semaphorin 3A blocks the formation of pathologic choroidal neovascularization induced by transforming growth factor beta. Mol Vis.

[168] Zehetner C, Kirchmair R, Neururer SB (2014). Systemic upregulation of PDGF-B in patients with neovascular AMD. Invest Ophthalmol Vis Sci.

